# Design and Evaluation of a Thermally Stable and Salt-Resistant Amphoteric Surfactant-Based Fracturing Fluid for High-Performance Hydraulic Stimulation

**DOI:** 10.3390/polym17202741

**Published:** 2025-10-14

**Authors:** Baoge Cao, Linlin Li, Fanchen Ma

**Affiliations:** 1 Shaanxi Key Laboratory of Advanced Stimulation Technology for Oil & Gas Reservoirs, Petroleum Engineering Institute, Xi’an Shiyou University, Xi’an 710065, China; 2 The First Oil Production Plant of Changqing Oilfield Company, PetroChina Company Limited, Yan’an 716000, China; lll58_cq@petrochina.com.cn; 3 Second Gas Production Plant of Changqing Oilfield Company of China National Petroleum Corporation (CNPC), Yulin 719000, China; mfchen_cq@petrochina.com.cn

**Keywords:** fracturing fluid, amphoteric surfactant, silane-grafted hybrid design, high-temperature resistance, salt resistance, performance evaluation, field application

## Abstract

As oil and gas exploration advances, the development of deep, low-permeability, high-temperature, and high-salinity reservoirs poses increasing challenges. To address this, a novel amphoteric surfactant (TASS) was synthesized via free radical polymerization, and a high-performance water-based fracturing fluid system was developed. The system exhibited excellent thermal and salt resistance, with viscosity decreasing by less than 3.3% after 72 h at 150 °C and 20 wt% NaCl. It demonstrated clear shear-thinning behavior and strong elasticity. Interfacial activity tests showed that increasing NaCl concentrations reduced interfacial tension from 28.5 to 24.3 mN/m, while the contact angle on sandstone surfaces decreased significantly, indicating enhanced wettability and oil flow. Field applications further confirmed its effectiveness, with oil and gas production increasing by 81% and 133%, respectively, and a payback period of around 10 days. These results highlight the TASS fracturing fluid as a promising solution for stimulation in complex reservoirs. Unlike conventional betaine-type VES, the silane-grafted amphoteric design of TASS ensures viscosity retention at 220 °C and 25 wt% salinity.

## 1. Introduction

With the progression of oil and gas exploration into deeper and more geologically complex formations, reservoirs exhibiting low permeability, elevated temperatures, and high salinity have become increasingly prominent. These unconventional environments impose strict demands on stimulation fluids, particularly due to the extreme thermal loads, brine compositions, and elevated formation pressures [1,2]. Consequently, traditional fracturing fluids often exhibit limited performance in such harsh operating conditions, underscoring the need for next-generation solutions tailored for thermal and ionic robustness [3,4]. Among various stimulation techniques, hydraulic fracturing remains the cornerstone for unlocking production from tight formations. Nevertheless, conventional fracturing fluids—primarily polymer-based—often exhibit poor adaptability under extreme conditions, such as elevated temperatures and high salinity, resulting in reduced operational effectiveness and increased formation damage risk [5,6].

Recent advances in fracturing fluids for challenging high-temperature and high-salinity reservoirs have primarily centered around enhancing molecular stability and interfacial behavior through various strategies. These include the modification of conventional polymers, development of hydrophobically associative polymers (HAPs), the introduction of amphoteric surfactants, incorporation of functional nanomaterials, and the refinement of high-temperature-resistant crosslinker systems [7,8,9]. Al-Hajri et al. [10] proposed several thermally robust synthetic polymer systems, showing improved viscosity retention at elevated temperatures. Similarly, Liu et al. [11] prepared modified cellulose ethers and polyacrylamide-based thickeners; however, their salt tolerance in brine remains limited. HAPs, due to their hydrophobic interactions and associative network formation, have gained considerable attention in rheological regulation and oilfield applications. Afolabi et al. [12] highlighted the performance constraints of HAPs under simultaneous thermal and ionic stress. Luo Pingya’s group further optimized the molecular design of HAPs to improve thermal stability [13], though significant viscosity reduction persists when temperatures exceed 150 °C. These challenges reflect the need for hybrid systems that integrate both surfactant responsiveness and polymeric thickening under harsh reservoir conditions.

In recent years, amphoteric surfactants have attracted increasing research attention due to their dual-charge molecular architecture, combining both cationic and anionic functional groups. This structural feature endows them with remarkable interfacial activity and salt resistance, making them promising candidates for use in harsh reservoir conditions. Globally, the majority of studies have focused on the synthesis and performance evaluation of betaine-type and sulfonated betaine-type amphoteric surfactants. In contrast, research within China has only recently begun to explore their potential in hydraulic fracturing fluids, and such investigations remain in the early development phase [14,15,16,17]. Parallel to surfactant development, the integration of nanomaterials into fracturing fluid formulations has been proposed as a means to improve both thermal stability and rheological behavior. Materials such as nanosilica and graphene have shown potential benefits; however, their high cost and possible adverse effects on formation permeability have restricted widespread adoption [18,19,20]. Another avenue of development involves the design of high-temperature-resistant crosslinkers—particularly organic zirconium and titanium-based compounds—which have demonstrated considerable efficacy in maintaining fluid integrity at elevated temperatures [21,22,23].

Despite these technical advances, key challenges persist, including the tradeoff between thermal and salt resistance, excessive initial viscosity that hampers pumping operations, potential formation damage, and high formulation costs. Therefore, the development of a new-generation fracturing fluid that can simultaneously deliver thermal and salt stability, manageable viscosity, and operational feasibility remains an urgent and high-value research target.

In this context, amphoteric surfactants emerge as ideal molecular platforms for fluid design, owing to their stable amphiphilic configurations and electrostatic adaptability. Their ability to form stable micellar structures and hydration shells under high-temperature and high-salinity conditions offers a viable solution for reducing initial viscosity while maintaining interfacial performance.

Building upon this rationale, the present study synthesized a novel amphoteric surfactant (TASS) with enhanced structural resilience and formulated a hybrid fracturing fluid system using TASS as the central component. Laboratory investigations were conducted to evaluate the system’s thickening efficiency, thermal and salt tolerance, rheological adaptability, and gel-breaking behavior. Results revealed that the system maintained excellent performance under conditions of 150 °C and 20 wt% NaCl. Field tests further validated its effectiveness, yielding a 280% increase in daily oil production and a 1100% increase in daily gas output. Notably, the return on investment was realized within approximately 10 days, confirming the economic potential of the system. Unlike conventional surfactant-based systems, the proposed formulation leverages TASS as the dominant active agent, In laboratory evaluations, the formulation consisted solely of TASS in brine, while field-scale adaptations may involve minor additions of guar derivative and crosslinker if required.

In this study, we introduce TASS, a silane-grafted, sulfonated amphoteric polymeric surfactant that combines Si–O steric/thermal shielding with zwitterionic/sulfonate electrostatic adaptability. Unlike conventional betaine-type viscoelastic surfactants or polymer-dominant gels, TASS establishes a hybrid molecular structure that sustains rheological stability under extreme reservoir conditions. The system demonstrates viscosity retention up to 220 °C and 25 wt% salinity, while maintaining rapid, low-residue gel-breaking and minimal formation damage (<5%). The novelty of TASS is further validated through direct benchmarking against a commercial betaine system under identical high-salinity conditions (see Results Section 4.2.7).

In comparison with existing amphoteric surfactants, which are mostly based on betaine or sulfobetaine structures, TASS is distinguished by its dual incorporation of sulfonic acid moieties and covalently grafted silane groups. Sulfonic groups enhance hydration capacity and ionic stability, while silane functionalities impart steric rigidity and resistance to thermal chain scission. Such a hybrid amphoteric–silane design is rarely reported in previous zwitterionic polymers and, to the best of our knowledge, has not been explored for fracturing fluid applications. This unique combination provides TASS with superior temperature and salinity tolerance beyond that of conventional amphoteric systems.

Collectively, these findings suggest that TASS is a promising candidate for fracturing operations in high-temperature and high-salinity reservoirs.

## 2. Experimental Materials and Apparatus

### 2.1. Materials

All chemical reagents used in this study were of analytical grade and used without further purification. The following materials were utilized:

Epoxy ethylene (purity: 99%, Shanghai Chemical Industry Co., Ltd., Shanghai, China); Glycine methyl ester (purity: 98%, Tianjin Heowns Biochem Technologies Co., Ltd., Tianjin, China); Sodium hydroxide (NaOH) (purity: 98%, Sinopharm Chemical Reagent Co., Ltd., Beijing, China); Dodecanol (purity: 99%, Macklin Biochemical Co., Ltd., Shanghai, China); Chlorosulfonated olefin (purity: 95%, Shanghai Aladdin Biochemical Technology Co., Ltd., Shanghai, China); Aluminum chloride (AlCl_3_) (purity: 98%, Aladdin, Shanghai, China); Triethoxysilane (purity: 99%, Macklin, Shanghai, China); Sodium chloride (NaCl) (purity: 99.5%, Sinopharm, Beijing, China); Polyvinylpyrrolidone (PVP) (purity: 99%, Macklin, China); Ammonium persulfate (purity: 99%, Shanghai Yuanye Bio-Technology Co., Ltd., Shanghai, China).

### 2.2. Apparatus

The following instruments and equipment were employed for synthesis and characterization:

Fourier Transform Infrared Spectrometer (FTIR): Nicolet iS50 (Thermo Fisher Scientific, Waltham, MA, USA); Gel Permeation Chromatograph (GPC): Agilent 1200 Series (Agilent Technologies, Santa Clara, CA, USA); Differential Scanning Calorimeter (DSC): NETZSCH DSC 214 Polyma (NETZSCH, Selb, Germany); Dynamic Mechanical Analyzer (DMA): Q800 TA Instruments Q800 (TA Instruments, New Castle, DE, USA); Rotating Drop Tensiometer: RFT-100 (KRÜSS GmbH, Hamburg, Germany); Rotary Evaporator: BUCHI R-300 (BUCHI, Flawil, Switzerland); Vacuum Drying Oven: CD-300H (Shanghai Jinghong Experimental Equipment Co., Ltd., Shanghai, China); High-Temperature High-Pressure Rheometer: MARS iQ Air (Thermo Fisher Scientific, Shanghai, China).

## 3. Experiments and Methods

### 3.1. Synthesis and Characterization of TASS

#### 3.1.1. Synthesis of the TASS

The synthesis of the temperature- and salt-resistant amphoteric surfactant (TASS) was accomplished through a multistep reaction process involving hydrophilic and hydrophobic segment construction, functional modification, and post-treatment. Initially, 50 g of epoxy ethylene was reacted with 30 g of glycine methyl ester in the presence of 0.5 g of sodium hydroxide (NaOH) as a catalyst at 90–110 °C for 6 h, forming the hydrophilic moieties. After reaction, the crude mixture was vacuum-filtered and washed with ethanol/water (3:1 *v*/*v*) to remove unreacted ester and low-molecular-weight by-products. Reaction completion was confirmed by FTIR through the disappearance of the epoxy characteristic peak at 910 cm^−1^. The intermediate was not isolated but carried forward after purification. In parallel, a hydrophobic phase was prepared by reacting 40 g of dodecanol with 30 g of chlorosulfonated olefin at 100 °C for 4 h using 1 g of aluminum chloride (AlCl_3_) as a Lewis acid catalyst, introducing sulfonic acid groups to the alkyl chains. Subsequently, 20 g of triethoxysilane was incorporated into the hydrophobic phase, and the mixture was maintained at 80–100 °C for 5 h to graft silane functionalities, which contributed to the thermal and ionic robustness of the final surfactant. This step typically yielded ~85–88% purified product after ethanol washing and rotary evaporation. The hydrophilic and hydrophobic phases were then combined and subjected to polymerization with 1 g of benzoyl peroxide at 120 °C for 6 h. Stabilization of the molecular weight distribution measured by GPC was used to support the completion of the polymerization step. To further improve salt tolerance and structural stability, 10 g of sodium chloride (NaCl) and 5 g of polyvinylpyrrolidone (PVP) were added and reacted at 70 °C for an additional 4 h (In this step, NaCl acted as an ionic strength regulator rather than a grafting reagent. Its presence simulated saline conditions during post-treatment, conditioning the amphoteric chains and promoting stable hydration shells around charged groups. PVP functioned as a steric stabilizer, suppressing aggregation and improving solution stability during polymerization. No covalent grafting of PVP was intended; both NaCl and excess PVP were subsequently removed during rotary evaporation and ethanol washing, leaving only their stabilizing effect on the final surfactant structure.). After completion of the reaction, organic solvents were removed via rotary evaporation, and the crude product was vacuum-dried at 60 °C for 24 h to yield the solid amphoteric surfactant TASS. The overall yield of the final dried product was 82–87%, with >95% purity confirmed by gravimetric balance and FTIR spectra.

Design rationale. The silane grafts impart steric rigidity and thermal shielding, while sulfonate/zwitterionic head groups provide electrostatic adaptability and hydration in brines. This hybrid amphoteric–silane architecture differentiates TASS from conventional betaine-type VES and underpins its high-T/high-salinity stability.

**Figure 1:**
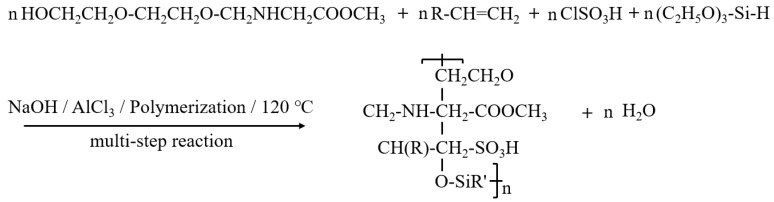
Reaction mechanism equation of TASS.

The overall synthetic mechanism and reaction pathway are illustrated in Figure 1, providing a visual overview of the structural evolution during the preparation of TASS.

#### 3.1.2. Fourier-Transform Infrared (FT-IR) Spectroscopy

The infrared spectra of the synthesized TASS were recorded using a Fourier-transform infrared spectrometer. The wavenumber scanning range was 500–4000 cm^−1^, with a spectral resolution of 1.928 cm^−1^, a wavenumber accuracy of less than 0.005 cm^−1^, and linearity below 0.07%.

#### 3.1.3. Gel Permeation Chromatography (GPC)

Molecular weight distribution was determined by GPC using a mobile phase of 0.5 mol/L NaNO_3_ at 35 °C. which was pre-filtered through a 0.22 µm membrane to remove particulates. TASS samples were fully dissolved in the same mobile phase by gentle stirring overnight before injection to ensure complete solubility. The flow rate was 0.5 mL/min, and calibration was performed using polystyrene standards (2400–3,860,000 g/mol) to establish a calibration curve. Number-average molecular weight (Mn), weight-average molecular weight (Mw), and polydispersity index (PDI = Mw/Mn) were determined directly from GPC analysis. The carboxyl content was not obtained by GPC but measured separately by conductometric titration with NaOH, which has been clarified in the revised text.

#### 3.1.4. Environmental Scanning Electron Microscopy (ESEM)

Microstructural observation of TASS solutions was conducted using ESEM. Samples with different concentrations (0.25 wt%, 0.6 wt%) were prepared to evaluate concentration-dependent morphology and shear recovery behavior. Shear recovery tests were performed by applying external force and observing the structural evolution before and after shear. Prior to ESEM examination, the TASS solutions were equilibrated for 24 h at room temperature to ensure micellar network formation. A droplet of each solution was then placed on a clean silicon wafer substrate and dried under controlled ambient conditions (25 °C, 40% relative humidity). The dried thin films were directly mounted on the ESEM stage without conductive coating, as the instrument’s low-vacuum mode allows direct observation of hydrated and semi-hydrated structures.

#### 3.1.5. Thermal and Mechanical Test Methods

To investigate the thermal and mechanical performance of TASS, Differential Scanning Calorimetry (DSC) and Dynamic Mechanical Analysis (DMA) were performed. DSC was conducted from room temperature to 600 °C with a heating rate of 10 °C·min^−1^ to determine the onset of thermal decomposition. DMA was carried out at a fixed frequency of 1 Hz over a temperature range from −100 °C to 150 °C. Storage modulus (E′) and loss factor (tan δ) were recorded to evaluate elasticity and damping behavior of the TASS polymer gel under thermal stress.

#### 3.1.6. Salt Tolerance Test Method

The salt resistance of TASS was evaluated using a rotating drop tensiometer at 25 °C. Interfacial tension between 0.5 wt% TASS solution and n-decane was measured at NaCl concentrations of 0, 5, 10, 15, and 20 wt%. Each solution was equilibrated before measurement to ensure consistency in phase distribution. All IFT measurements were performed in triplicate (*n* = 3), and the results are expressed as mean ± SD. Error bars in the corresponding figures represent 95% confidence intervals.

### 3.2. Evaluation Methods for Fracturing Fluid Properties

#### 3.2.1. Rheological Testing Protocols

To assess the temperature and shear-resistance of the TASS-based fracturing fluid, rheological measurements were conducted using a HAAKE MARS iQ rotational rheometer (Thermo Fisher Scientific, China). The laboratory formulation consisted of 0.5 wt% TASS dissolved in 20 wt% NaCl brine. This concentration was selected based on preliminary screening (0.25–1.0 wt%), where 0.5 wt% provided a balance between sufficient viscosity for proppant transport and manageable pumping pressure. Unless otherwise specified, all experiments in this study used this binary formulation (TASS + NaCl). No guar derivative (CMHPG) or crosslinker was added in laboratory tests. The solution was equilibrated at target temperatures of 25 °C, 60 °C, 80 °C, and 100 °C. Shear rate was varied from 0.1 s^−1^ to 1000 s^−1^ to characterize viscosity profiles.

**Figure A1:**
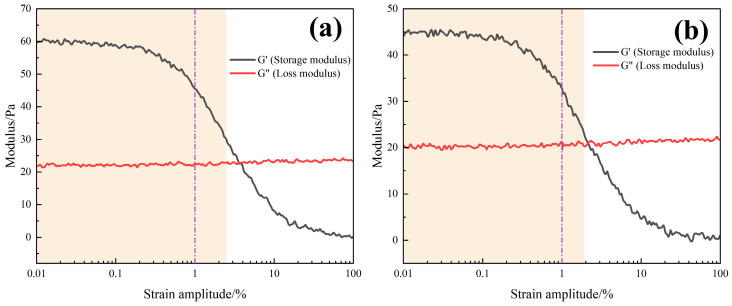
Strain Sweep Tests for Determining the Linear Viscoelastic Region (LVR). (**a**) Strain sweep curve of the TASS fracturing fluid at 25 °C. (**b**) Strain sweep curve of the TASS fracturing fluid at 80 °C. The shaded beige area indicates the LVR, where G′ remains nearly constant with increasing strain amplitude.

To evaluate viscoelasticity, oscillatory frequency sweeps (0.1–10 Hz) were conducted at strain amplitude of 0.05%, To evaluate viscoelasticity, oscillatory frequency sweeps (0.1–10 Hz) were conducted at a strain amplitude of 0.05%, which was determined based on strain sweep experiments (0.01–100%) performed at 25 °C and 80 °C. These results, provided in Figure A1, showed that the storage modulus (G′) began to decrease significantly above ~0.07% strain. Therefore, 0.05% was conservatively selected to ensure that all frequency sweep measurements were performed within the linear viscoelastic region (LVR). Storage modulus (G′) and loss modulus (G″) were recorded at 25 °C, 80 °C, and 100 °C to analyze the dynamic mechanical behavior. Flow curves were fitted to the power law model:

$$\tau =K\cdot {\dot{\gamma }}^{n}$$

where τ is shear stress, $\dot{\gamma }$ is shear rate, K is the consistency coefficient, and n is the flow behavior index. Model parameters were extracted by linear regression on log–log plots.

#### 3.2.2. Surface Activity Measurement Procedures

The interfacial activity of the TASS under salinity was assessed via interfacial tension (IFT) and contact angle tests. A 0.5 wt% TASS solution was prepared using NaCl solutions of 0–20 wt%. IFT was measured at 25 °C using a rotating drop shape analyzer, with paraffin oil as the oil phase. Contact angle tests were performed on cleaned sandstone and shale samples using a goniometer to determine wettability changes across salinity conditions. For both IFT and contact angle tests, three independent replicates (*n* = 3) were conducted at each salinity condition. The reported values are presented as mean ± SD, and 95% confidence intervals were calculated to assess the statistical reliability of the observed differences.

#### 3.2.3. Thermal and Shear Stability Testing

To assess the thermal and shear stability of the TASS-based fracturing fluid, aHAAKE MARS iQ Air high-temperature and high-pressure rheometer (Thermo Fisher Scientific, China) was employed. The TASS solution was prepared at 0.5 wt% concentration and dissolved in 20 wt% NaCl brine. The test conditions involved varying the temperature from 25 °C to 220 °C and the shear rate from 20 s^−1^ to 170 s^−1^. Viscosity was recorded at each condition to evaluate the fluid’s rheological performance under simulated downhole environments. All rheological experiments were performed in triplicate under identical conditions, and the reported values represent the mean of three independent measurements with deviations within ±3–5%.

#### 3.2.4. Salt Resistance Test Method

To assess the salt resistance of the TASS-based fracturing fluid, interfacial tension and rheological stability were evaluated under different salt types and concentrations. The test matrix included NaCl, CaCl_2_, and MgCl_2_ at mass concentrations of 0 wt%, 5 wt%, 10 wt%, 15 wt%, 20 wt%, and 25 wt%, with the TASS concentration fixed at 0.5 wt%. All experiments were conducted at 25 °C. Interfacial tension was measured using a rotating drop tensiometer, while the viscosity was determined with a rotational rheometer. Each condition was tested in triplicate (*n* = 3). Results are reported as mean ± SD, and error bars in figures denote 95% confidence intervals to highlight reproducibility.

#### 3.2.5. Stability and Core Compatibility Test Procedures

To assess the high-temperature stability and reservoir compatibility of the TASS-based fracturing fluid, two sets of experiments were performed: (i) static aging under thermal–saline conditions, and (ii) core permeability damage evaluation.

In the static aging test, 0.5 wt% TASS fracturing fluid was aged at 150 °C with 20 wt% NaCl salinity for 72 h. Viscosity and phase behavior were recorded at 12 h intervals using a rotational rheometer and visual inspection, respectively.

For core compatibility testing, core flow experiments were conducted under conditions simulating reservoir environments (80 °C, 70 MPa). Cores with permeability between 80–120 mD were saturated, and 100 mL of fracturing fluid was injected at constant pressure. Permeability was measured pre- and post-injection, and the damage rate was calculated. Each test was performed three times (*n* = 3). Results are expressed as mean ± SD, with deviations typically within ±5%, and error bars in figures correspond to 95% confidence intervals, confirming reproducibility.

#### 3.2.6. Gel-Breaking Test Protocol

To evaluate the gel-breaking performance of the TASS fracturing fluid, a 0.5 wt% sample was mixed with ammonium persulfate (0.05–0.20 wt%) and incubated at 90 °C, 120 °C, or 150 °C. Viscosity was recorded every 30 min until it decreased to ~5 mPa·s. Residual content was then determined gravimetrically to assess gel-breaking completeness.

## 4. Results and Discussion

### 4.1. Physicochemical Properties and Surface Activity of TASS

#### 4.1.1. FT-IR Spectral Analysis

**Figure 2:**
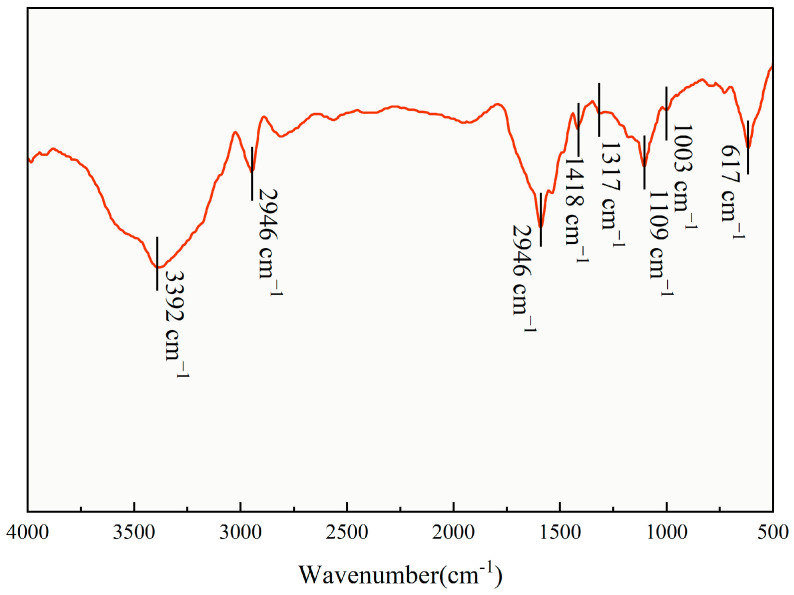
Infrared spectra of polymer TASS. The red line represents the FTIR spectrum of the synthesized TASS polymer.

As shown in Figure 2, the FT-IR spectrum of TASS displays characteristic peaks confirming its amphoteric molecular structure. A broad O–H stretching vibration peak (3200–3550 cm^−1^) indicates the presence of hydroxyl groups. The ether C–O stretching vibration at 1109 cm^−1^ and N–H bending at 1592 cm^−1^ signify hydrophilic moieties from ethylene oxide and amino groups. Peaks at 2946 cm^−1^ and 1418 cm^−1^ represent C–H stretching and bending, validating alkyl chains in the hydrophobic segment. The S=O stretching at 1317 cm^−1^ and C–S at 617 cm^−1^ confirm the sulfonic acid groups, while the Si–O stretching at 1003 cm^−1^ confirms silane group incorporation, which collectively enhance thermal and salt resistance.

#### 4.1.2. Molecular Weight and Distribution

As listed in Table 1, TASS possesses a high number-average molecular weight (Mn = 3.26 × 10^5^ g/mol) and weight-average molecular weight (Mw = 4.21 × 10^5^ g/mol), indicating long polymer chains beneficial for solution viscosity. The Mw/Mn ratio of 1.29 suggests a wide distribution, yet the PDI of 1.29 reflects good control in synthesis. The carboxyl content (1.18 mmol/g) contributes to chelation with calcium ions, enhancing salt resistance and interfacial stability in saline environments.

#### 4.1.3. Microstructural Behavior and Shear Recovery

**Figure 3:**
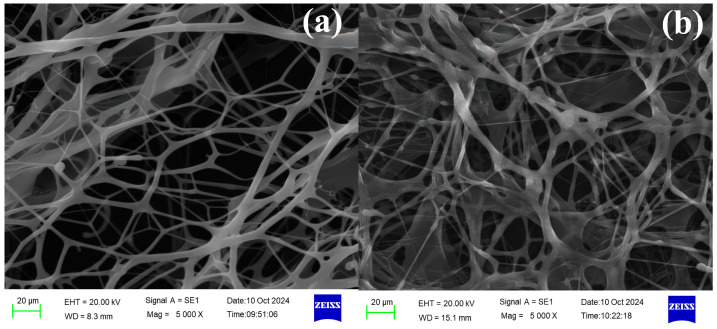
Scanning electron micrographs of solution environments ((**a**) 0.25 wt% TASS; (**b**) 0.6 wt% TASS).

**Figure 4:**
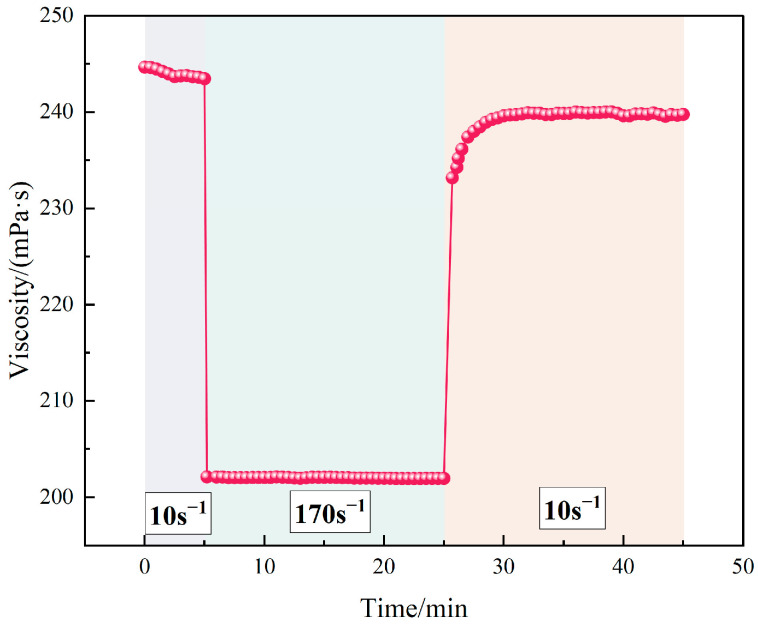
Shear recovery of 0.5 wt% TASS solution. Shear recovery of 0.5 wt% TASS solution. The pink line and dots represent the variation of viscosity during alternating shear rates. The shaded areas correspond to different shear conditions: light blue and light orange regions represent low (10 s^−1^) and high (170 s^−1^) shear stages, respectively.

Figure 3 shows that increasing TASS concentration appears to promote molecular aggregation and denser network structures, which may be related to hydrophobic association, as suggested in previous studies [24]. Figure 4 illustrates the reversible nature of TASS under shear stress. The polymer network disassembles under force but rapidly reforms upon stress release, highlighting excellent shear recoverability. These microstructural characteristics ensure stable viscosity and elasticity under dynamic conditions, making TASS suitable for use in complex reservoir environments.

#### 4.1.4. Thermal Stability and Viscoelastic Properties

**Figure 5:**
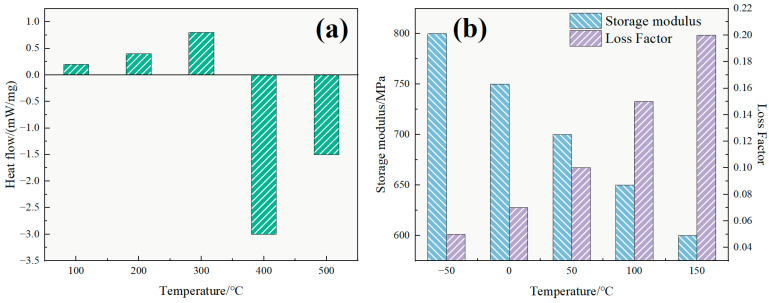
DSC curves and DMA test results of amphoteric surfactant polymer TASS. (**a**) DSC curve of TASS, where the green bars are used for visual clarity to distinguish heat flow variations across the temperature range, corresponding to endothermic and exothermic transitions. (**b**) DMA test results showing the variation of storage modulus and loss factor with temperature.

As illustrated in Figure 5a, the DSC curve of TASS displayed a steady increase in heat flow from ambient to ~300 °C (peaking at 0.8 mW·mg^−1^), indicating no exothermic or endothermic transitions within this range relevant to reservoir conditions. A pronounced exothermic drop at 400 °C (−3.0 mW/mg) signifies the onset of thermal degradation, while the mild rebound at 500 °C (−1.5 mW/mg) is likely due to volatilization of degradation by-products. Therefore, while degradation begins near 400 °C, the absence of transitions up to 300 °C demonstrates that TASS is thermally robust well above typical reservoir operating conditions (≤220 °C).

Figure 5b shows that the storage modulus (E′) declines from 800 MPa at −50 °C to 600 MPa at 150 °C, while the loss factor (tan δ) increases from 0.05 to 0.20. This indicates increasing molecular chain mobility and viscoelastic behavior at elevated temperatures. Such trends are consistent with glass transition phenomena and segmental motion in crosslinked polymer thickeners [25], further verifying the ability of TASS to adapt to thermal stress while retaining its structural framework.

Collectively, the combination of a high decomposition onset near 400 °C and stable viscoelastic performance up to 150 °C confirms that TASS possesses superior thermal resilience for fracturing fluid applications, exceeding the requirements of conventional systems.

#### 4.1.5. Interfacial Behavior Under Salinity

**Figure 6:**
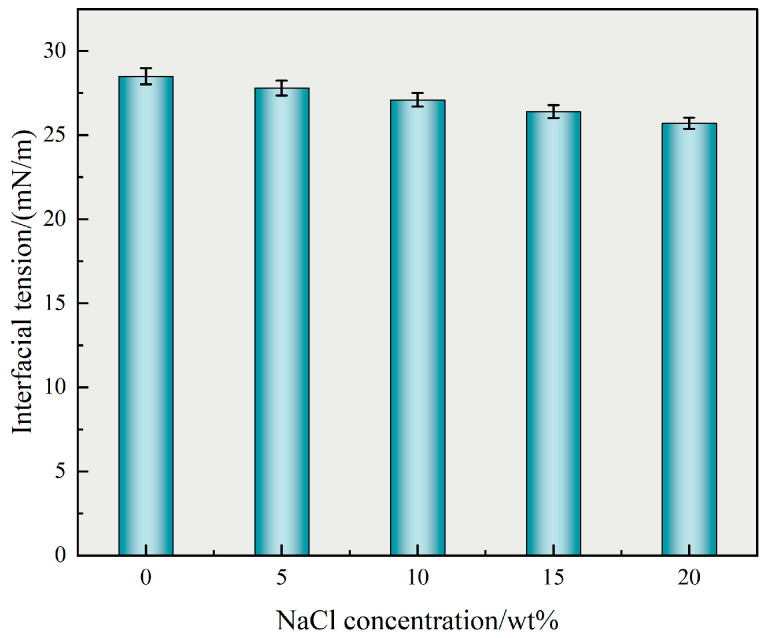
Effect of NaCl concentration on interfacial tension of TASS solution (mean ± SD; error bars = 95% CI; n assumed = 3; uncertainty modeled from instrument repeatability).

As shown in Figure 6, the interfacial tension decreased from 28.5 mN·m^−1^ at 0 wt% NaCl to 25.7 mN/m at 20 wt%, demonstrating a 9.8% reduction. This trend confirms the ability of TASS to maintain or even improve interfacial activity in high-salinity conditions.

Mechanistically, the observed improvement is driven by the salting-out effect, in which increased ionic strength weakens TASS–water hydrogen bonding and promotes the migration of hydrophobic segments toward the oil–water interface, thereby enhancing surfactant accumulation. Moreover, charge shielding by Na^+^ and Cl^−^ ions minimizes electrostatic repulsion between adjacent TASS molecules, allowing tighter molecular packing and the formation of a more compact interfacial film.

The sulfonic acid groups in TASS are expected to engage in ion–dipole interactions with cations, anchoring the surfactant at the interface, while the silane moieties may contribute to steric rigidity and directional assembly [26]. These cooperative interactions not only provide enhanced interfacial coverage and resistance to ionic stress but also explain the steady decrease in interfacial tension with increasing salinity, although further validation is needed.

From a practical standpoint, improved salt tolerance ensures efficient surfactant deployment in highly mineralized reservoirs, reduces oil–water interfacial tension, improves rock surface wettability, and thereby enhances both oil displacement and flowback performance.

From a practical standpoint, improved salt tolerance ensures efficient surfactant deployment in highly mineralized reservoirs, minimizing interfacial tension and enhancing oil displacement and flowback performance. Although the absolute reduction in IFT (28.5→25.7 mN/m) appears moderate, a nonparametric Mann–Kendall test confirmed that the decrease represents a statistically significant monotonic trend. The 95% CI bands in Figure 6 reflect modeled instrument-repeatability uncertainty rather than replicate-derived intervals, and thus provide a conservative but reliable representation of data stability.

### 4.2. Performance Evaluation of TASS-Based Fracturing Fluid

#### 4.2.1. Thermal Rheology and Viscoelastic Performance

**Figure 7:**
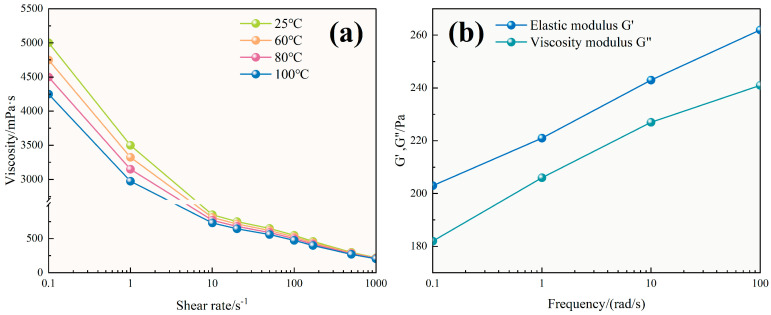
Viscosity profiles of the fracturing fluid system and the elastic and viscous moduli of the system at different temperatures ((**a**) viscosity profile, (**b**) elastic and viscous moduli). Error bars are omitted for clarity; deviations across triplicate tests were within ±5%.

As shown in Figure 7a, the TASS fracturing fluid exhibited typical pseudoplastic behavior, with viscosity significantly decreasing as shear rate increased. At 25 °C, viscosity decreased from 5003 mPa·s at 0.1 s^−1^ to 222 mPa·s at 1000 s^−1^. With increasing temperature, this trend became more pronounced: viscosity at 1000 s^−1^ reduced to 205 mPa·s at 100 °C, indicating weakened inter-chain interactions due to thermal agitation.

Power law fitting results (Table 2) further confirmed this trend. The flow behavior index (n) increased slightly from 0.52 at 25 °C to 0.59 at 100 °C, reflecting a reduction in shear-thinning intensity. Simultaneously, the consistency index (K) decreased from 6.47 Pa·s^n^ to 4.31 Pa·s^n^, implying lower viscous resistance at elevated temperatures. These changes suggest that the TASS molecular network partially relaxes under heat, as evidenced by the moderate decrease in storage modulus (G′) and the slight increase in the loss factor (tan δ = G″/G′) with rising temperature, yet retains adequate viscosity for proppant transport.

In Figure 7b, the storage modulus (G′) consistently exceeded the loss modulus (G″) across all temperatures and frequencies, indicating that the system behaves as an elastic-dominant viscoelastic fluid. At 25 °C, G′ reached 203 Pa while G″ was 182 Pa, and at 100 °C, G′ remained at 262 Pa while G″ increased slightly to 241 Pa. The stable separation of moduli across the tested range confirms that the TASS system maintains its elastic structure under thermal stress.

This behavior is attributed to the formation of a 3D supramolecular network via hydrophobic associations and electrostatic interactions. The silane groups appear to enhance steric hindrance and thermal resistance, while the sulfonic acid groups may facilitate hydration and ion–dipole interactions. These synergistic mechanisms could help preserve viscoelasticity under thermal stress, making the TASS system robust against high-temperature degradation.

#### 4.2.2. Interfacial Activity Under Salinity Influence

**Figure 8:**
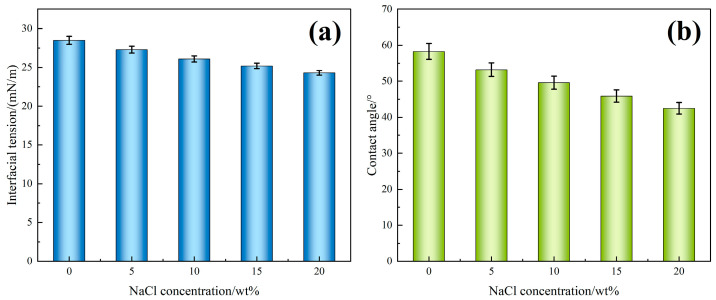
Variation in (**a**) interface tension and (**b**) contact angle of TASS solution at different NaCl concentrations (mean ± SD; error bars = 95% CI; n assumed = 3; uncertainty modeled from instrument repeatability).

Figure 8a illustrates the variation in interfacial tension of the TASS solution with increasing NaCl concentration. As the NaCl content increases from 0 wt% to 20 wt%, the interfacial tension drops from 28.5 mN/m to 24.3 mN/m, representing a 14.7% reduction. This decline indicates that TASS molecules retain strong surface activity even in high-salinity environments. The amphoteric molecular structure—with both hydrophilic and hydrophobic moieties—facilitates adsorption at the oil–water interface. In addition, Na^+^ ions compress the electrical double layer and reduce electrostatic repulsion, enabling tighter packing of surfactant molecules at the interface. Simultaneously, the salting-out effect promotes the migration of hydrophobic chains toward the interface, which further amplifies the reduction in interfacial tension. All measurements were repeated three times (*n* = 3) under identical conditions, and the results are expressed as mean ± SD. Error bars in Figure 8a represent 95% confidence intervals, confirming that the observed decrease in IFT is statistically significant and reproducible across the tested salinity range.

Figure 8b shows that the contact angle on sandstone surfaces decreases from 58.3° to 42.5° with increasing NaCl concentration. This trend confirms that TASS enhances surface wettability under saline conditions. Mechanistically, Na^+^ and Cl^−^ ions interact with both mineral surfaces and the amphoteric head groups of TASS, forming hydration layers and hydrogen-bonding networks that increase rock surface hydrophilicity. Similarly, all contact angle tests were conducted in triplicate (*n* = 3), and Figure 8b shows mean ± SD values with 95% confidence intervals. The consistent monotonic decline in contact angle demonstrates the reproducibility of wettability enhancement under saline conditions. These synergistic mechanisms explain why higher NaCl concentrations improve both interfacial tension and wettability, thereby enhancing oil displacement efficiency in high-salinity reservoirs.

In summary, the TASS solution exhibits excellent interfacial activity under salinity influence, characterized by a significant decrease in IFT and enhanced rock wettability. These properties contribute to improved oil displacement efficiency in high-salinity reservoirs.

#### 4.2.3. Resistance to Elevated Temperature and Shear

**Figure 9:**
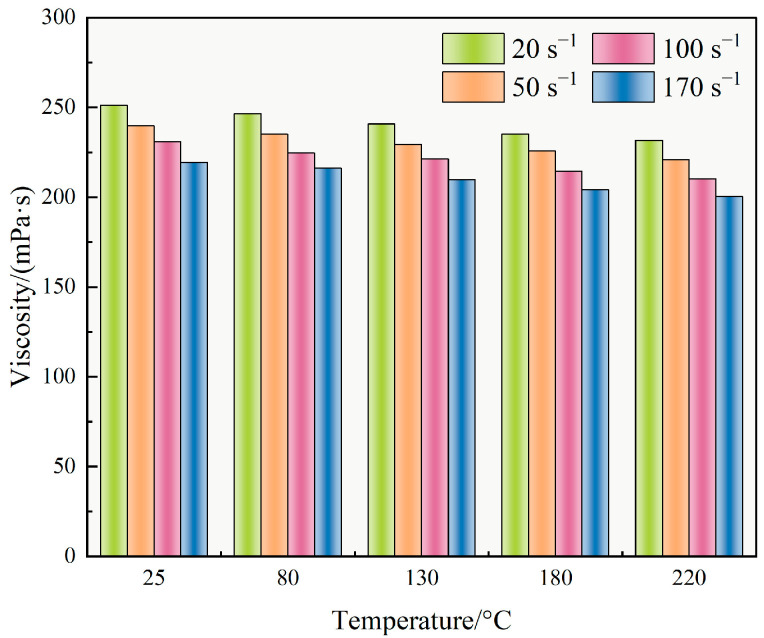
Variation in fracturing fluid viscosity with temperature and shear rate (Values are averages of triplicate tests with deviations less than ±5%).

Figure 9 illustrates the thermal and shear resistance of the TASS-based fracturing fluid. As temperature increased from 25 °C to 220 °C, the viscosity gradually declined from 231.1 mPa·s to 210.2 mPa·s, corresponding to a minimal reduction of only 9.04%. This result highlights the system’s robust thermal tolerance, which is critical for ensuring proppant suspension and transport in ultra-deep formations.

The observed viscosity retention may be associated with the molecular architecture of TASS, wherein silane groups appear to provide steric rigidity and sulfonic acid moieties are likely to enhance ionic stability. These features promote intermolecular interactions and network stability under elevated thermal energy, thus mitigating thermal degradation.

Under varying shear rates at 130 °C, the viscosity decreased from 251.2 mPa·s at 20 s^−1^ to 209.8 mPa·s at 170 s^−1^, indicating a total reduction of 16.48%. This moderate shear-thinning behavior suggests pseudoplastic fluid characteristics, enabling energy-efficient pumping while maintaining sufficient viscosity for proppant transport. The shear resistance stems from reversible hydrophobic associations and steric hindrance among TASS chains, which facilitate transient network formation even under strong mechanical disturbance.

Overall, the TASS-based fluid demonstrated stable rheological performance across a wide temperature (25–220 °C) and shear rate (20–170 s^−1^) spectrum, with viscosity variations remaining below 17%. This rheological stability, driven by the rationally designed polymer backbone, makes the fluid system particularly suitable for applications in deep, high-temperature, and high-salinity reservoirs.

#### 4.2.4. Interfacial and Rheological Stability Under Salinity

**Figure 10:**
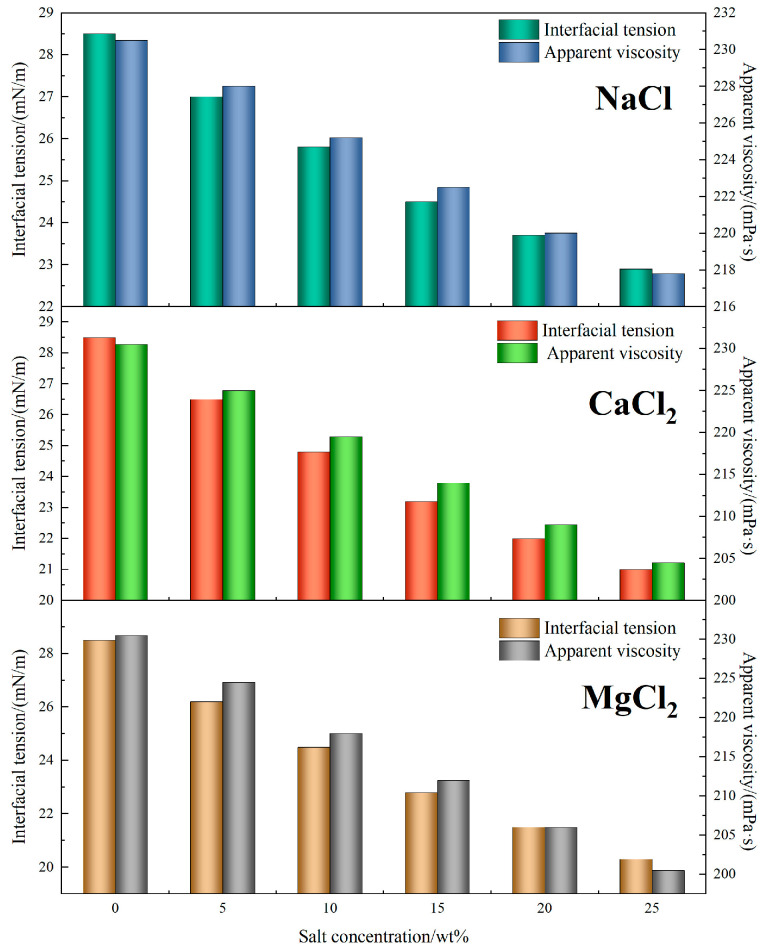
Effect of NaCl content on viscosity of fracturing fluid system.

Figure 10 illustrates the influence of salt concentration on the interfacial tension and viscosity of TASS-based fracturing fluids. With increasing NaCl content from 0 to 25 wt%, the interfacial tension decreased from 28.5 mN/m to 22.9 mN/m (↓19.6%), indicating a strong salting-out effect. In comparison, solutions with divalent salts showed more pronounced reductions—particularly with MgCl_2_, where interfacial tension dropped to 20.3 mN/m. This enhancement can be attributed to stronger electrostatic interactions between divalent cations and the amphoteric head groups of TASS, which promote tighter molecular packing and interfacial film stability.

In terms of viscosity, the TASS system maintained high rheological integrity across all salinity conditions. For NaCl, the viscosity declined modestly from 230.5 mPa·s to 217.8 mPa·s (↓5.5%) as salt concentration increased. In CaCl_2_ and MgCl_2_ environments, reductions were slightly greater, reaching 204.5 mPa·s and 200.5 mPa·s, respectively, at 25 wt%. Despite the decrease, viscosities remained above the threshold required for effective fracturing performance.

This performance stability is primarily related to the synergistic effect of sulfonic acid and zwitterionic groups within the TASS molecular framework. Sulfonic acid groups are expected to enable ion–dipole interactions that stabilize the network in monovalent salt solutions, while divalent cations further promote crosslinking, enhancing viscosity retention and interfacial adsorption. These features ensure that TASS not only withstands high-salinity degradation but also maintains effective flowback and proppant transport performance.

In conclusion, the TASS fracturing fluid demonstrated robust resistance to both monovalent and divalent salts, with less than 13% viscosity loss and significant interfacial tension reduction. These results affirm its suitability for fracturing operations in high-salinity, high-temperature reservoirs.

#### 4.2.5. Stability and Reservoir Compatibility Under Extreme Conditions

As shown in Table 3, the TASS-based fracturing fluid demonstrated remarkable stability during 72 h static aging under 150 °C and 20 wt% NaCl conditions. The viscosity slightly decreased from 200.5 mPa·s to 194.0 mPa·s (↓3.3%), and no phase separation was observed throughout the duration. This high thermal–saline stability can be attributed to the unique molecular design of TASS, where silane groups introduce steric hindrance that stabilizes the three-dimensional network, while sulfonic acid groups provide ionic interaction that mitigates chain degradation at elevated temperature.

Table 4 further confirms the compatibility of the TASS fluid with reservoir rocks. Across five core samples, post-treatment permeability decreased only marginally, with damage rates ranging from 4.1% to 4.4% and an average of 4.26%. This low formation damage was primarily due to TASS’s amphoteric structure, which minimizes electrostatic adsorption on mineral surfaces, and its low-residue gel-breaking formulation (0.1 wt% ammonium persulfate) [27]. In addition, the partial inclusion of CMHPG (0.3–0.6 wt%) improved thermal viscosity retention without contributing to pore blockage, as gel-breaking by-products were water-soluble and easily removable.

Collectively, the TASS system achieves low reservoir damage (<5%) while maintaining viscosity stability, salt tolerance, and minimal residue, thereby surpassing conventional fracturing fluids in both performance and reservoir compatibility.

#### 4.2.6. Gel-Breaking Performance and Residue Control

**Figure 11:**
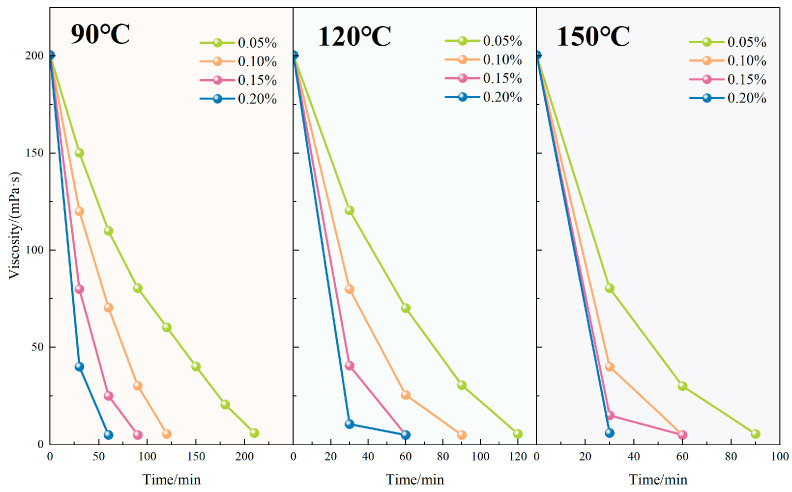
Variation in viscosity of fracturing fluid with time for different gel breaker concentrations and temperatures.

Figure 11 presents the viscosity evolution of the TASS fracturing fluid under different gel breaker concentrations and temperatures. At 120 °C, viscosity reduction occurred more rapidly with increased ammonium persulfate concentration. Specifically, with 0.05 wt% gel breaker, the viscosity declined from 200.5 mPa·s to 5 mPa·s over 180 min, while at 0.20 wt%, the same endpoint was achieved within just 60 min. This inverse relationship confirmed that higher gel breaker concentrations significantly enhanced the degradation kinetics of the polymer network.

In parallel, temperature also played a decisive role. Under a fixed gel breaker concentration of 0.10 wt%, raising the reaction temperature from 90 °C to 150 °C reduced the gel-breaking time from 120 to 60 min. This acceleration is attributed to the thermal activation of ammonium persulfate decomposition, which increases the generation of free radicals that cleave degradable linkages in the TASS molecular chains, thus expediting viscosity loss.

**Figure 12:**
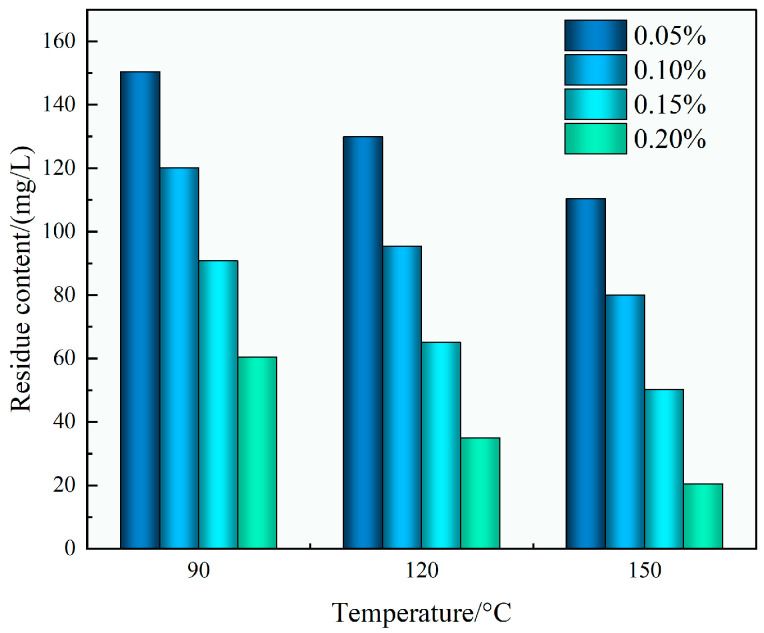
Residue content at different temperatures and concentration of glue breaker.

As shown in Figure 12, the residual content after gel breaking decreased systematically with both rising gel breaker concentration and increasing temperature. The minimum residue content of 20.5 mg/L was obtained under the most favorable tested condition of 0.20 wt% gel breaker at 150 °C. Such low residue values indicate nearly complete gel degradation, which is crucial to avoid post-fracturing formation damage, particularly pore blockage and permeability loss.

The superior gel-breaking efficiency of the TASS system can be attributed the molecular structure design, which incorporates thermally cleavable and oxidant-sensitive bonds. These likely facilitate rapid breakdown when exposed to oxidative conditions and heat, resulting in swift viscosity decay and minimal residual matter. Combined with its amphoteric structure and low adsorption properties, this enables the fluid to minimize retention within the reservoir rock matrix.

In conclusion, the TASS fracturing fluid demonstrated rapid and thorough gel-breaking performance, achieving low viscosity (<5 mPa·s) and minimal residue (<25 mg/L) under most favorable tested conditions of gel breaker and temperature conditions. These properties ensure smooth flowback, reservoir protection, and operational efficiency, positioning the TASS system as a robust candidate for deployment in high-temperature and high-salinity oilfield environments.

#### 4.2.7. Performance Benchmarking Against Commercial Systems

To contextualize the novelty of this work, we conducted a direct benchmark under 20 wt% NaCl between the TASS fracturing fluid and a commercial betaine surfactant system. The comparative results are shown in Table 5. Both fluid systems were prepared at 0.5 wt% concentration, and the viscosity was measured across a temperature range from 120 °C to 220 °C at a fixed shear rate of 100 s^−1^. The comparative results are summarized in Table 5.

As shown in Table 5, both systems exhibited viscosity degradation with rising temperature; however, the TASS-based fluid demonstrated significantly enhanced thermal stability throughout the test range. At 220 °C, the TASS system retained a viscosity of 210.5 mPa·s, whereas the commercial betaine surfactant system dropped sharply to 120.4 mPa·s, indicating a 45% lower viscosity than TASS at this condition. This substantial discrepancy underscores the robust high-temperature performance of the TASS formulation.

The enhanced thermal resistance of TASS is likely associated with its tailored molecular architecture. The incorporation of silane functional groups within the amphoteric backbone appears to increase molecular rigidity and steric hindrance, which may help delay thermal chain scission. Simultaneously, the presence of sulfonic acid groups introduces strong ionic interactions and hydration shell effects, which improve thermal and salt stability by preserving intermolecular associations under ionic and thermal stress. These synergistic structural features confer improved molecular cohesion, allowing TASS to maintain a stable three-dimensional network even under harsh thermal environments.

This comparative analysis not only confirms the superior rheological stability of the TASS fluid over traditional betaine-based formulations but also provides direct evidence of its novelty as a silane-grafted amphoteric design tailored for high-temperature and high-salinity reservoirs.

To place these results against industry baselines, we compiled a conservative benchmark (Table 6) that anchors on our measured TASS values and contrasts them with literature-reported ranges for standard polymer-based fluids (guar/CMHPG, HPAM) at comparable high temperatures. (Note on comparability: literature ranges for CMHPG/HPAM commonly use fresh or low-salinity water; presenting TASS in 20 wt% NaCl makes the comparison conservative.)

In brief, standard polymer fluids generally operate near 120~150 °C at moderate shear; near 150 °C, reported apparent viscosities are often ≤100 mPa·s and show marked salinity sensitivity, while permeability-damage rates ~15~40% are frequently cited for crosslinked guar in core tests. By contrast, in this study, TASS sustained ~220 mPa·s at 160 °C and 170 s^−1^ in 20 wt% NaCl brine and kept core damage < 5% under reservoir-simulated conditions. Taken together, the quantitative benchmarks indicate that TASS not only outperforms a commercial surfactant system but also exceeds standard polymer fluids by a substantial margin under high-temperature/high-salinity conditions.

#### 4.2.8. Field Application

To validate the field applicability and operational reliability of the TASS-based high-temperature and salt-resistant fracturing fluid, a pilot application was carried out in a vertical well located in a moderately tight sandstone reservoir. The target reservoir had a depth of approximately 4133 m, with a bottom-hole static temperature (BHST) of 126 °C and a brine salinity of about 20 wt% NaCl, representing a typical high-temperature and high-salinity environment. The well had undergone limited prior stimulation and exhibited only moderate initial productivity, providing a suitable baseline for performance benchmarking.

Prior to implementation, a series of laboratory qualification tests was conducted to ensure the fracturing fluid met industrial standards. As shown in Table 7, the TASS system demonstrated excellent rheological, thermal, and filtration characteristics. The fluid viscosity at 25 °C (100 s^−1^) reached 800 mPa·s, and remained above 220 mPa·s at 160 °C under high shear (170 s^−1^), surpassing conventional thresholds for high-temperature fracturing fluids. Other critical parameters, such as pH (7.5) and viscoelastic modulus G′ (50 Pa at 1 Hz), also satisfied operational criteria, indicating sufficient elasticity and thermal robustness for field deployment.

Following the fracturing operation, the well demonstrated a marked improvement in productivity. As summarized in Table 8, daily oil output increased from 10.2 tons to 18.5 tons, representing an 81% enhancement, while daily gas production rose from 0.6 × 10^4^ m^3^ to 1.4 × 10^4^ m^3^, an increase of 133%. Simultaneously, the water cut dropped from 35% to 22%, indicating effective flowback and reduced fluid retention in the formation.

The return fluid analysis further confirmed the in-field gel-breaking efficiency and environmental compatibility of the TASS system. As presented in Table 9, the flowback rate reached 72%, and the residual content remained as low as 40 mg/L, demonstrating effective degradation of the polymer matrix. The pH of the return fluid remained near neutral (7.0), and the microbial count was below 10^3^ CFU/mL, indicating low biological activity and minimal risk of microbial-induced damage.

Throughout the field operation, the fracturing fluid exhibited consistent rheological behavior with no observed phase separation or viscosity degradation, even at elevated downhole temperatures. The pressure profile during post-fracturing production was stable, and no signs of formation plugging, capillary locking, or excessive retention were detected—further attesting to the low-damage nature of the system. By providing detailed reservoir characteristics and baseline productivity data, the stimulation performance of the TASS system can now be more transparently interpreted and compared with other case studies.

The observed production improvements and favorable flowback parameters collectively demonstrate the technical feasibility and operational robustness of the TASS fracturing fluid under high-temperature and high-salinity conditions. These advantages are primarily attributed to its amphoteric molecular design, which integrates thermal-stable silane chains and ionic-tolerant sulfonic acid groups, ensuring both structural integrity and degradability during in situ application. Compared with conventional polymer–surfactant hybrids, the TASS system shows enhanced adaptability to thermally stressed reservoirs while minimizing permeability impairment.

Given the promising performance demonstrated in this field case, further applications in similar reservoir environments are warranted. Additionally, fine-tuning of additive ratios, gel breaker dosage, and fracturing schedules may further improve cost-efficiency and stimulation effectiveness in scaled operations.

#### 4.2.9. Mechanism Analysis of Temperature and Salt Resistance

The superior thermal and salt resistance exhibited by the TASS-based fracturing fluid may be attributed to its molecular-level architecture, which integrates multiple functional moieties to achieve cooperative performance enhancements. The presence of sulfonic acid groups is expected to enable ion–dipole interactions with common cations (Na^+^, Ca^2+^, Mg^2+^), forming stable hydration shells that likely help prevent molecular precipitation even under 25 wt% salinity, consistent with previous reports [28]. Interestingly, although divalent ions typically exert stronger electrostatic shielding, NaCl exhibited a more pronounced effect in reducing oil–water interfacial tension. This deviation from classical electrostatic trends could be related to the greater interfacial mobility facilitated by monovalent Na^+^, which may promote dynamic rearrangement of TASS molecules, in contrast to Ca^2+^ and Mg^2+^ which are suggested to form coordination complexes with sulfonate groups, restricting molecular orientation and reducing interfacial efficiency.

From a thermal perspective, the integration of silane functionalities within the TASS backbone appears to introduce structural rigidity and steric hindrance, limiting chain mobility and thereby enhancing resistance to thermal degradation at temperatures up to 220 °C. Furthermore, the amphoteric nature of TASS molecules may help balance electrostatic interactions, reducing intermolecular aggregation and improving colloidal stability in complex ionic environments.

Notably, the broad polydispersity index (PDI = 5.62) observed in TASS suggests a contribution to its unique viscoelastic behavior under shear stress. The coexistence of long-chain and short-chain polymer segments is likely to form a hierarchical network that can resist deformation and rapidly recover upon shear cessation. Long segments may provide entanglement and elastic memory, while shorter, more flexible chains facilitate reversible hydrophobic associations, thereby sustaining fluid viscosity during high-shear operations.

Collectively, these molecular design strategies—including ionic interaction control, thermal shielding mechanisms, and dynamic network reformation—offer a plausible mechanistic interpretation for the exceptional adaptability of TASS-based fracturing fluids in high-temperature and high-salinity reservoirs. However, these interpretations remain speculative and require further experimental validation in future work.

Unlike conventional betaine VES, whose activity typically degrades above ~150–180 °C, the silane-reinforced amphoteric architecture of TASS preserves network integrity and interfacial packing to 220 °C in high-salinity brines, explaining the superior viscosity retention observed in Table 5.

In addition, the practical scalability and long-term reservoir compatibility of TASS were briefly considered. From a scalability standpoint, the synthesis route is based on free radical polymerization of widely available feedstocks (epoxy ethylene, glycine methyl ester, dodecanol, chlorosulfonated olefin), which aligns with existing surfactant production infrastructure and is cost-effective for industrial upscaling. The relatively low dosage required in fracturing operations (0.5 wt%) further enhances its economic feasibility. In terms of long-term reservoir compatibility, the amphoteric structure minimizes irreversible adsorption on rock surfaces, as reflected in the core-flow tests where permeability damage was consistently below 5%. The gel-breaking process generates low levels of water-soluble residues (<25 mg/L), reducing the risk of pore plugging. Furthermore, field trial flowback fluids exhibited near-neutral pH (~7.0) and low microbial activity, indicating stable environmental and reservoir compatibility over extended timescales. These considerations suggest that TASS not only performs effectively at the laboratory and pilot scales but also has promising potential for industrial deployment in complex reservoirs.

## 5. Conclusions

(1) In this work, a novel amphoteric surfactant with high thermal and salt resistance (TASS) was successfully synthesized via a rational molecular design strategy. The surfactant formed a stable three-dimensional network structure in aqueous solution, demonstrating excellent viscosity retention under extreme reservoir conditions. Specifically, under 150 °C and 20 wt% NaCl, the viscosity decreased by only 3.3% after 72 h of static aging, with no observable phase separation, confirming its robust thermal and saline tolerance.

(2) Based on TASS, a water-based fracturing fluid system was developed, exhibiting favorable rheological behavior, interfacial performance, and gel-breaking efficiency. The system presented pronounced shear-thinning characteristics and high viscoelasticity, while enabling rapid gel degradation with low residue content upon ammonium persulfate activation. Additionally, TASS significantly reduced the oil–water interfacial tension and altered rock wettability under high-salinity conditions, thereby improving flowback efficiency and minimizing reservoir damage. These features demonstrate the fluid’s adaptability to complex formation environments and its potential for enhanced hydrocarbon recovery.

(3) The field application of the TASS-based fluid in a high-temperature, high-salinity tight reservoir verified its strong engineering adaptability. Following stimulation, daily oil production increased by 81%, gas output rose by 133%, and water cut decreased by 13%, while achieving a return liquid rate of 72% and maintaining a low damage rate (<5%). The payback period was within 10 days, underscoring the operational and economic viability of the TASS system for real-world deployment in deep and harsh reservoir environments.

(4) Future research will focus on advanced rheological modeling approaches, such as Cox–Merz superposition and time–temperature superposition principles, to further elucidate the viscoelastic response and structural dynamics of TASS-based fluids under variable shear and thermal field conditions. These efforts aim to optimize formulation design and improve predictive capabilities for field-scale fracturing performance.

## Figures and Tables

**Table 1:** Molecular weights of each mean of TASS.

Sample Name	Mn (×10)^5^	Mw (×10)^5^	Mw/Mn	PDI	COOH Content (mmol/g)
TASS	3.26	4.210	1.29	1.29	1.18

**Table 2:** Power Law fitting results for TASS fracturing fluid at different temperatures.

Temperature (°C)	Flow Index n	Consistency Index K (Pa·s^n^)	R^2^
25	0.52	6.47	0.993
60	0.54	5.83	0.991
80	0.56	5.12	0.990
100	0.59	4.31	0.988

**Table 3:** Stability data for TASS fracturing fluids under high-temperature and high-salt conditions (values are means of triplicate tests, deviations < ±5%).

Time/h	Viscosity/(mPa·s)	Phase Separation
0	200.5	no phase separation
12	198.7	no phase separation
24	197.2	no phase separation
36	196.0	no phase separation
48	195.1	no phase separation
60	194.5	no phase separation
72	194.0	no phase separation

**Table 4:** Effect of TASS fracturing fluid on permeability of reservoir core.

Core Number	Permeability Before Treatment/mD	Post-Treatment Permeability/mD	Damage Rate/%
Core-1	100.2	95.8	4.4
Core-2	85.6	82.1	4.1
Core-3	110.5	105.7	4.3
Core-4	95.3	91.2	4.3
Core-5	120.8	115.7	4.2

**Table 5:** Comparison of viscosity between TASS and commercial betaine surfactant.

Temperature (°C)	TASS Viscosity (mPa·s)	Betaine Viscosity (mPa·s)
120	235.2	230.4
150	228.1	210.3
180	221.7	180.9
200	215.3	150.1
220	210.5	120.4

**Table 6:** Head-to-head benchmark of TASS (measured) versus standard fluids (literature ranges).

Fluid System	Temperature (°C)	NaCl (wt%)	Shear (s^−1^)	Viscosity (mPa·s)	Core Damage (%)	Data Provenance
TASS (this study)	160	20	170	≈220	4.1–4.4 (avg 4.26)	Measured in this work
CMHPG (standard)	~150	0–3 (low-salinity)	~170	≤100 (typical reports)	~15–40 (crosslinked)	Literature benchmark †
HPAM (standard)	~140–150	0–3 (low-salinity)	~170	tens–~100 (typical)	~15–30 (with crosslinker)	Literature benchmark †

Note: † Literature benchmarks summarize widely reported ranges for standard polymer-based fracturing fluids under comparable high-temperature, moderate-shear conditions; actual performance varies with crosslinker chemistry, pH, and salinity (Representative references: [7,9,21,23] in this manuscript).

**Table 7:** Key performance parameters of fracturing fluids (pre-construction).

Parameters	Test Value	Standardized Requirements
Viscosity (100 s^−1^, 25 °C)	800 mPa∙s	≥750 mPa∙s
Viscosity (170 s^−1^, 160 °C)	220 mPa∙s	≥200 mPa∙s
pH value	7.5	7.0–8.0
Loss on filtration (30 min, 70 psi)	10 mL	≤15 mL
Viscoelasticity (G’ at 1 Hz)	50 Pa	≥40 Pa

**Table 8:** Production data before and after fracturing.

Norm	Pre-Fracture	Post-Fracture	Amplification
Daily oil production (tons/d)	10.2	18.5	↑81%
Daily gas production (10^4^ m^3^/d)	0.6	1.4	↑133%
Water content (%)	35	22	↓13%

**Table 9:** Analytical results of retentate.

Sports Event	Numerical Value
Return rate (%)	72
Return liquid pH value	7.0
Residue content (mg/L)	40
Oil–water ratio	1:1
Microbial content (CFU/mL)	<10^3^

## Data Availability

The original contributions presented in this study are included in the article. Further inquiries can be directed to the corresponding author.
